# Liver Damage in Patients with HCV/HIV Coinfection Is Linked to HIV-Related Oxidative Stress

**DOI:** 10.1155/2016/8142431

**Published:** 2016-01-10

**Authors:** Xiangbo Huang, Hua Liang, Xueying Fan, Liyan Zhu, Tao Shen

**Affiliations:** ^1^Department of Microbiology & Infectious Disease Center, School of Basic Medical Sciences, Peking University, Beijing 100191, China; ^2^State Key Laboratory of Infectious Disease Prevention and Control, National Center for AIDS/STD Control and Prevention, China CDC, Collaborative Innovation Center for Diagnosis and Treatment of Infectious Diseases, Beijing 102206, China

## Abstract

HIV infection aggravates the progression of liver damage in HCV-coinfected patients, with the underlying pathogenesis being multifactorial. Although high level of oxidative stress has been observed frequently in patients infected with HIV or HCV, the status of oxidative stress in HIV/HCV coinfection and its contribution to HCV liver damage have not been determined. This study involved 363 HBsAg-negative, anti-HCV-positive former blood donors recruited from a village in central China in July 2005; of these, 140 were positive for HIV. Of these 363 subjects, 282 were successfully followed up through July 2009. HIV/HCV-coinfected subjects had higher rates of end-stage liver disease-related death than those monoinfected with HCV. Liver ultrasound manifestations were poor in HIV-positive than in HIV-negative individuals, in both chronic HCV carriers and those with resolved HCV. Serum concentrations of total glutathione (tGSH), malondialdehyde (MDA), glutathione peroxidase (GSH-Px), GSSG, and reduced GSH were higher in HIV-positive than HIV-negative subjects. GSSG concentrations were higher in HIV-infected subjects with abnormal ALT/AST levels than in those with normal ALT/AST levels and were associated with poorer liver ultrasound manifestations. These finding indicated that HIV infection accelerated HCV-associated liver damage in HIV/HCV-coinfected individuals. Increased oxidative stress, induced primarily by HIV coinfection, may contribute to aggravated liver damage.

## 1. Introduction

Although most individuals exposed to hepatitis C virus (HCV) develop chronic infection, about one quarter to one-third of HCV-infected subjects show spontaneous viral clearance [1, 2]. HCV and human immunodeficiency virus (HIV) share similar routes of transmission, particularly through contaminated blood or blood products. For example, contaminated blood and plasma collection practices in central China in the early 1990s resulted in high rates of HCV and HIV infections among rural farmer donors and more than 90% of HIV-infected subjects coinfected with HCV [3–10]. Hepatotoxicity in HIV-1 monoinfection strongly correlates with CD4+ counts [11] and HIV coinfection can aggravate HCV-related liver damage [7] via mechanisms that include losses of CD4+ T helper cells and neutralizing antibodies [12], dysfunction of dendritic cells (DCs) [13], iron overload [14], and possibly oxidative stress.

Glutathione is a tripeptide with a gamma peptide linkage between the carboxyl group of the glutamate side-chain and the amine group of cysteine; this molecule serves as a key antioxidant, preventing damage to important cellular components caused by reactive oxygen species, such as free radicals and peroxides [15]. Glutathione is present in both reduced (GSH) and oxidized (glutathione disulfide (GSSG)) forms. Glutathione reduces disulfide bonds within cytoplasmic proteins to cysteine residues by serving as an electron donor. During this process, reduced GSH is converted to its oxidized form, GSSG [16]. Furthermore, glutathione peroxidase (GSH-Px), a component of the GSH cycle, is one of the most important antioxidant enzymes in humans, catalyzing the breakdown of lipid peroxides and hydroperoxides in both extracellular and intracellular compartments and protecting the integrity of membranes against peroxidative interference and damage [16, 17]. Malondialdehyde (MDA) [18], an important biomarker of oxidative stress, is generated by the peroxidation of lipids containing polyunsaturated fatty acids and is cytotoxic due to its ability to induce macromolecular crosslinking polymerization.

*In vitro* studies have shown that oxidative stress is increasing during both HIV [19] and HCV [20] infection. Long-term oxidative stress can lead to an overabundance of free radicals in the liver, contributing to liver fibrosis, cirrhosis, and carcinogenesis. Evaluations of individuals monoinfected with HIV and coinfected with HIV/HCV showed that oxidative stress was greater and levels of serum antioxidants were lower in HIV/HCV-coinfected subjects than in HIV-monoinfected subjects, which is consistent with the observation that fibrosis scores were higher in coinfected subjects compared to HIV monoinfection [19, 21]. These finding hinted that higher oxidative stress status in coinfected subjects was at least partially contributed to HCV infection. However, whether HIV-induced oxidative stress is linked to faster liver disease progression in HIV/HCV-coinfected patients still lacks evidence. We speculated that long-term oxidative stress induced by HIV infection may lead to overabundant free radicals in liver and contribute to liver damage. This study investigated the possible relationship between higher concentrations of circulating free radicals and aggressive liver damage in HIV/HCV-coinfected patients by analyzing the serum levels of oxidants and antioxidants.

## 2. Materials and Methods

### 2.1. Study Participants

This study involved 363 HBsAg-negative, anti-HCV-positive former blood donors (FBDs) recruited from a village in central China in July 2005, including 140 coinfected with HIV. In a follow-up survey in July 2009, 282 patients were successfully visited, 36 had died, and 45 were lost to follow-up. Serum concentrations of anti-HCV and anti-HIV antibodies and of HCV and HIV RNA were measured. Subjects positive for anti-HIV antibody were defined as HIV-positive. Subjects positive for anti-HCV antibody and with detectable HCV RNA were defined as chronically infected with HCV, whereas subjects positive for anti-HCV antibody but negative for HCV RNA on two separate occasions at least six months apart were defined as spontaneously clearing HCV. Based on these definitions, the 282 participants were divided into four groups, 102 HIV-negative chronic HCV carriers, 76 HIV-positive chronic HCV carriers, 56 HIV-negative HCV resolvers, and 48 HIV-positive HCV resolvers. A flow diagram of the subjects for this study is shown in Figure S1 (see Supplementary Material available online at http://dx.doi.org/10.1155/2016/8142431). None of the participants received any form of HCV-specific antiviral therapy, whereas all HIV-positive subjects received regular or intermittent highly active antiretroviral therapy (HAART). HAART regimens included two nucleoside reverse transcriptase inhibitors (NRTIs), either azidothymidine plus didanosine or stavudine plus lamivudine, and one nonnucleoside reverse transcriptase inhibitor (NNRTI), nevirapine. The control group consisted of 18 healthy adults negative for HIV, HBV, and HCV infection.

All participants were interviewed by trained and qualified staff using a standardized questionnaire to collect general information, blood donation history, and use of antiviral or antiretroviral drugs. The demographic and clinical characteristics of all participants are shown in Table S1. All subjects were assessed by routine blood tests and serum biochemical tests.

The study was approved by the institutional review authorities of Peking University Health Science Center (Approval ID: PKUPHLL20090011). All patients provided written informed consent before enrollment in the study.

### 2.2. Liver Ultrasound Examination

Fasting liver ultrasound examinations were performed using a Convex Ultrasound Scanner (GE LOGIQ 9, GE Medical System, CA, USA). Grey-scale images of the liver were obtained using a 3.5–5 MHz multifrequency transducer. Ultrasonography was performed by two senior physicians, each with more than 10 years of clinical experience. Ultrasound results were classified as normal, altered echostructure, hepatomegaly, diffuse liver parenchyma lesions, and fatty liver. Altered echostructure was defined as intrahepatic hyperechoic and heterogeneous echotexture. Diffuse liver parenchyma was considered an independent factor and was not included in the group with altered echostructure. Fatty liver and its severity were diagnosed according to the criteria from the American Gastroenterology Association, including (1) a diffuse hyperechoic echotexture, (2) increased liver echotexture compared with the kidneys, (3) vascular blurring, and (4) deep attenuation [22].

### 2.3. HIV and HCV Seropositive Screening and Confirmation

Plasma anti-HCV antibody was detected using Abbott Architect anti-HCV assays (Abbott GmbH & Co. KG, Wiesbaden, Germany) and confirmed by HCV-RIBA assays (Wantai Biological Pharmacy, Beijing, China). HIV infection was screened by ELISA with HIV antibodies (GBI Biotech Co., Ltd., Beijing, China) and confirmed by HIV Blot 2.2 WB assays (Genelabs Diagnostics, Singapore).

### 2.4. Quantification of HCV-RNA, HIV-RNA, and CD4+/CD8+ T-Cell Counts

Plasma HCV RNA concentrations were measured using the Abbott Real-Time HCV Amplification Kit (Abbott Molecular Inc. Des Plaines, IL, USA), according to the manufacturer's instructions; the limit of detection was 30 IU/mL. Plasma concentrations of HIV-1 RNA were measured with the Standard Amplicor HIV Monitor assay, version 2.0 (Roche Diagnostics, Indianapolis, IN, USA), according to the manufacturer's protocols; the limit of detection was 40 copies/mL.

CD4+ T-cell counts were measured by staining EDTA-treated whole blood for CD3/CD4/CD8/CD45 in TruCount tubes and analyzing with a FACSCalibur flow cytometer (Becton Dickinson, San Jose, CA, USA). The absolute numbers of CD4+ T lymphocytes were determined using MultiSET software (BD Bioscience, San Jose, CA, USA).

### 2.5. Measurements of Oxidative Stress

Three types of glutathione (total and the reduced and oxidized forms) were measured using GSH colorimetric detection kits (Cayman Chemical, Ann Arbor, MI, USA) after treatment with 2-vinylpyridine. Serum GSH-Px and MDA concentrations were measured with colorimetric detection kits (Jiancheng, Nanjing, China) according to the manufacturer's instructions. Serum concentrations of zinc were measured by the deproteinization method using a Perkin-Elmer 503 atomic absorption spectrophotometer.

### 2.6. Liver Fibrosis Stages

Stages of liver disease and liver fibrosis were measured using the aspartate aminotransferase to platelet ratio index (APRI) and the FIB-4 fibrosis index [23]. APRI was calculated according to the following formula: AST [IU/L]/(upper limit of normal range) × 100/platelet count (10^9^/L). The upper limit of the normal range in this study was 40 IU/L. FIB-4 index was calculated using a formula that included patient age, serum aspartate aminotransferase (AST) and alanine aminotransferase (ALT) concentrations, and platelet counts [24]; that is, FIB-4 index = (age [years] × AST [IU/L])/(platelet count [10^9^/L] × ALT [IU/L]^1/2^).

### 2.7. Statistical Analyses

All statistical analyses were performed using GraphPad Prism for Windows, version 5.0 (GraphPad Software Inc., San Diego, CA). Continuous variables were compared using unpaired *t*-tests or Mann-Whitney *U* tests. Categorical variables were compared using Pearson Chi-squared tests, including characteristics of HCV and HIV infection, HCV-related mortality rates, liver echostructure, HIV status, and serum GSSG. Correlations between groups were evaluated by Spearman correlation analysis. All tests were two-tailed, and *P* values <0.05 were considered statistically significant.

## 3. Results

### 3.1. Mortality from 2005 to 2009

Of the 363 HCV-infected participants recruited in 2005, 36 died between 2005 and 2009, including seven HCV-monoinfected and 29 HIV/HCV-coinfected patients. The mortality rate was significantly higher in coinfected than in HCV-monoinfected patients (20.7% versus 3.14%, *P* < 0.001). End-stage liver diseases (ESLDs) were responsible for 66.7% (8/12) of non-AIDS-related deaths in HIV/HCV-coinfected subjects, but only 14.3% (1/7) of deaths in HCV-monoinfected subjects (*P* = 0.027) (Table 1).

### 3.2. Poorer Liver Ultrasound Manifestations in HIV-Coinfected Subjects

Of the 363 subjects recruited in 2005, 282 (77.7%) were followed up and underwent liver ultrasound examinations in 2009. Ultrasound manifestations of poorer liver histology were observed in HIV-positive compared with HIV-negative individuals, both for chronic HCV carriers and those who experienced spontaneous resolution (*P* < 0.001 each; Table 2). Diffuse liver parenchyma lesions were more common in HIV-positive than in HIV-negative individuals chronically infected with HCV (39.5% versus 2.0%) and in those who showed HCV resolution (12.5% versus 0%). Altered echostructure was also more frequent in HIV-positive than in HIV-negative individuals with HCV resolution (54.2% versus 28.6%). The frequencies of hepatomegaly and fatty liver were similar in HIV-positive and HIV-negative individuals, both in chronic HCV carriers and in HCV resolvers.

### 3.3. Higher Serum tGSH, MDA, and GSH-Px Concentrations and Lower Zinc Concentrations, in HIV-Coinfected Subjects

**Figure 1:**
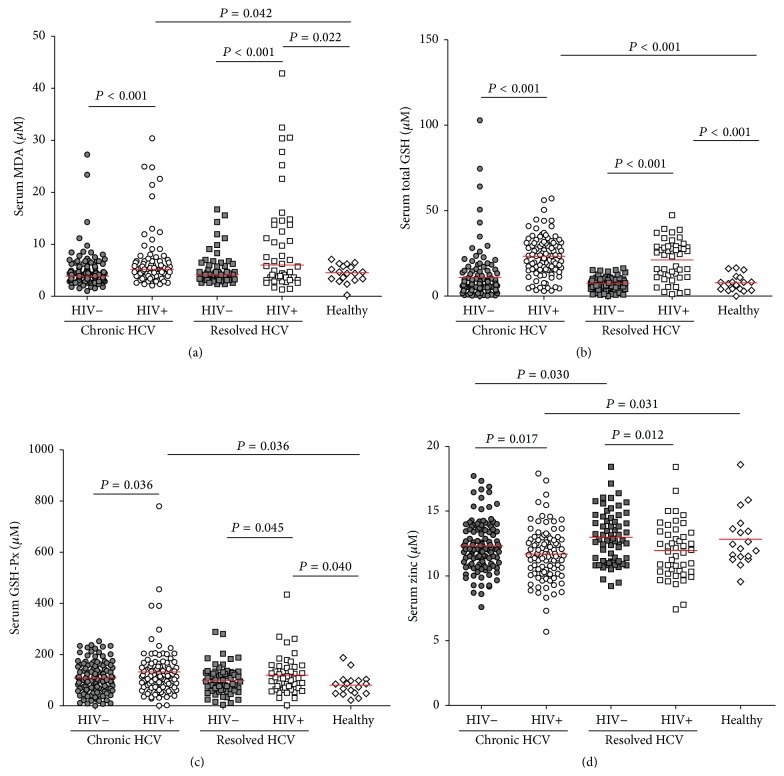
Serum concentrations of the oxidative stress markers MDA (a), total GSH (b), GSH-Px (c), and zinc (d) in HIV-negative chronic HCV carriers (●) (*N* = 102), HIV-positive chronic HCV carriers (○) (*N* = 76), HIV-negative HCV resolvers (■) (*N* = 56), HIV-positive HCV resolvers (□) (*N* = 48), and healthy controls (⋄) (*N* = 18). The median value for each group is indicated as a red line. Unpaired *t*-tests or Mann-Whitney *U* tests were used for between-group comparisons. All *P* values were two-tailed, with *P* < 0.05 considered statistically significant.

Oxidative stress levels in monoinfected and coinfected subjects were analyzed by measuring serum concentrations of tGSH, MDA, and GSH-Px and zinc concentrations (Figure 1). Overall, HIV-infected patients, both chronic HCV carriers and HCV resolvers, had significantly higher concentrations of MDA (*P* = 0.042 and *P* = 0.022, resp.), total GSH (*P* < 0.001 each), and GSH-Px (*P* = 0.036 and *P* = 0.040, resp.) than healthy controls. Serum concentrations of tGSH (*P* < 0.001), MDA (*P* < 0.001), and GSH-Px (*P* = 0.036) were significantly higher in HIV-positive than in HIV-negative HCV carriers. Similarly, serum concentrations of tGSH (*P* < 0.001), MDA (*P* < 0.001), and GSH-Px (*P* = 0.045) were significantly higher in HIV-positive than in HIV-negative HCV resolvers (Figures 1(a), 1(b), and 1(c)). By contrast, there were no differences in serum tGSH, MDA, and GSH-Px concentrations between chronic HCV carriers and spontaneous resolvers, either in the presence or absence of HIV coinfection. Lower serum zinc concentrations were observed in HIV-positive than in HIV-negative subjects, both in chronic HCV carriers (*P* = 0.017) and HCV resolvers (*P* = 0.012). Moreover, serum zinc concentrations differed significantly in HIV-negative chronic HCV carriers and resolvers (*P* = 0.030, Figure 1(d)).

### 3.4. Higher GSSG Was Induced by Either HIV or HCV Infection and Lower CD4+ T-Cell Counts Were Related to Lower Reduced GSH in HIV-Infected Patients

**Figure 2:**
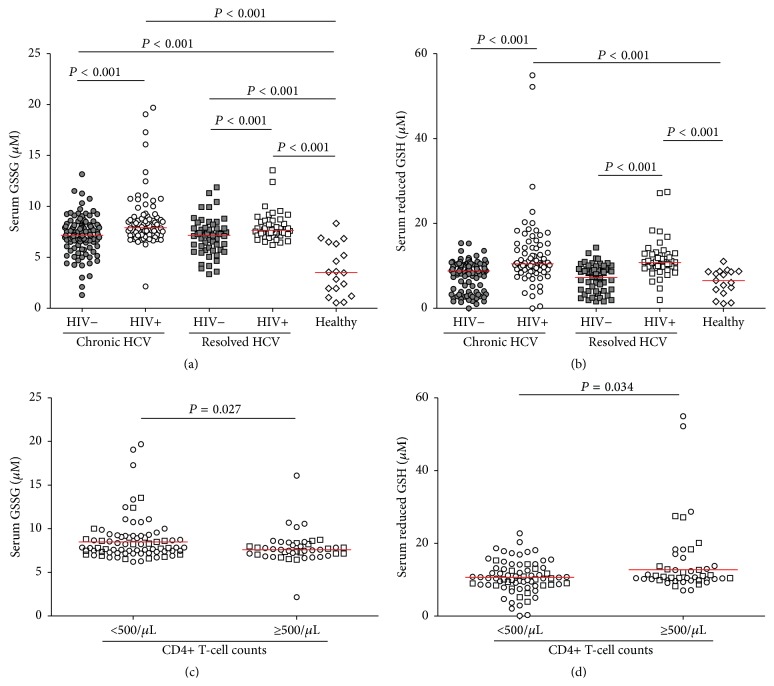
Serum concentration of GSSG (a) and reduced GSH (b) in HIV-negative chronic HCV carriers (●), HIV-positive chronic HCV carriers (○), HIV-negative HCV resolvers (■), HIV-positive HCV resolvers (□), and healthy controls (⋄). Serum concentrations of GSSG (c) and reduced GSH (d) in HIV-positive chronic HCV carriers (○) and HIV-positive HCV resolvers (□) were compared in stratified group according to CD4+ T-cell counts (<500/*μ*L versus ≥500/*μ*L). Median value for each group was indicated as red line. Unpaired *t*-tests or Mann-Whitney *U* tests were used for between-group comparisons. All *P* values were two-tailed, with *P* < 0.05 considered statistically significant.

Figure 2(a) shows the concentrations of reduced GSH and GSSG across the five groups of subjects. GSSG concentration was significantly higher in all groups of HCV- and/or HIV-infected subjects than in healthy controls (*P* < 0.001 each), as well as being significantly higher in HIV-positive than in HIV-negative subjects, both for chronic HCV carriers and HCV resolvers (*P* < 0.001 each). No differences were observed between chronic HCV carriers and spontaneous resolvers, either in the presence or absence of HIV coinfection, indicating that oxidative stress remained in the livers of subjects with resolved HCV infection, despite viral clearance.

The concentration of reduced GSH was calculated by subtracting the concentration of GSSG from that of total GSH. Reduced GSH concentration was significantly higher in HIV-positive than in HIV-negative subjects, both among chronic carriers and resolvers, and healthy controls (*P* < 0.001 each, Figure 2(b)). These results indicated that the profile of serum reduced GSH was different than that of GSSG, since HIV infection increased GSSG and reduced GSH concentrations, whereas HCV infection increased only GSSG concentration.

In addition, HIV-infected patients (HIV-positive chronic HCV carriers and HIV-positive HCV resolvers) were stratified according to immune status (CD4+ T-cell counts <500/*μ*L and CD4+ T-cell counts ≥500/*μ*L). Analysis showed that HIV-infected patients with lower CD4+ T-cell counts had significantly higher GSSG (*P* = 0.027, Figure 2(c)) while lower reduced GSH (*P* = 0.034, Figure 2(d)) concentrations than patients with higher CD4+ T-cell counts.

### 3.5. Serum GSSG Was Elevated in HIV-Infected Patients with Abnormal ALT/AST Levels

**Figure 3:**
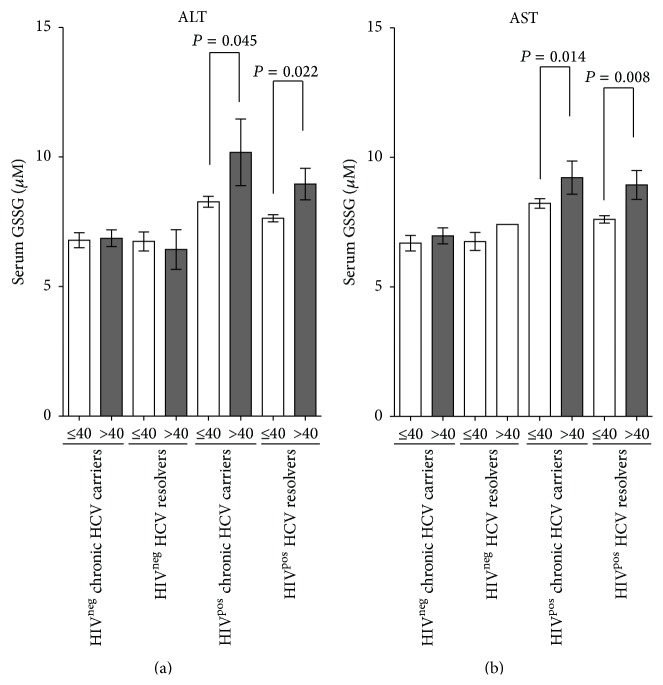
Serum GSSG concentrations are higher in HIV-infected patients with abnormal than in those with normal ALT/AST. Mean ± standard error of the mean (SEM) serum GSSG concentrations in HIV-negative chronic HCV carriers, HIV-positive chronic HCV carriers, HIV-negative HCV resolvers, HIV-positive HCV resolvers, and healthy controls with normal (≤40 IU/L) and abnormal (>40 IU/L) serum ALT (left panel) or AST (right panel). Unpaired nonparametric *t*-tests were used for between-group comparisons. Average and SEM were indicated for each group. All *P* values were two-tailed, with *P* < 0.05 considered statistically significant.

Serum concentrations of AST and ALT were significantly higher in HCV-monoinfected subjects than in those who experienced spontaneous recovery from HCV (*P* < 0.001 each, Figure S2). Similarly, higher serum AST (*P* < 0.001) and ALT (*P* = 0.060) concentrations were observed in HIV-positive compared to HIV-negative HCV resolvers (Figure S2). Although neither ALT nor AST showed significant correlations with serum GSSG concentrations in both HCV-monoinfected and HIV/HCV-coinfected patients (data not shown), HIV infection of subjects with abnormal (>40 IU/L) AST and ALT levels was associated with significant increases in serum GSSG, both in chronic HCV carriers (*P* = 0.045 for ALT and *P* = 0.014 for AST) and in HCV resolvers (*P* = 0.022 for ALT and *P* = 0.008 for AST) (Figure 3).

### 3.6. Serum Level of GSSG Was Associated with Poorer Liver Ultrasound Manifestations in HIV-Positive Individuals

Chronic HCV-infected patients may develop liver fibrosis, with some progressing to cirrhosis and even to hepatocellular carcinoma. To evaluate the effects of HIV coinfection on liver fibrosis, APRI and FIB-4 scores were calculated. Compared with HCV resolvers, chronic HCV carriers, whether HIV-negative or HIV-positive, had higher APRI (*P* = 0.004 and *P* = 0.007, resp.) and FIB-4 (*P* = 0.028 and *P* = 0.050, resp.) scores (Figure S3 a and b). In addition, HIV-positive individuals, both chronic HCV carriers and resolvers, had significantly higher APRI (*P* = 0.008 and *P* = 0.002, resp.) and FIB-4 (*P* = 0.014 and *P* = 0.027, resp.) scores than HIV-negative subjects. Analysis of APRI and FIB-4 scores in different liver ultrasound manifestations (normal, altered echostructure, and diffuse liver parenchyma lesions) in HIV-positive patients showed that APRI score was higher in patients with altered echostructure (*P* = 0.031) and diffuse liver parenchyma (*P* = 0.005) and that FIB-4 score was higher in patients with diffuse liver parenchyma (*P* = 0.017) (Figure S3 c and d). Additionally, negative correlations were found between CD4 T-cell counts and APRI (*P* = 0.037)/FIB-4 (*P* = 0.038) in HIV-positive HCV resolvers (Figure S4 b), but not in HIV-positive chronic HCV carriers (Figure S4 a).

**Figure 4:**
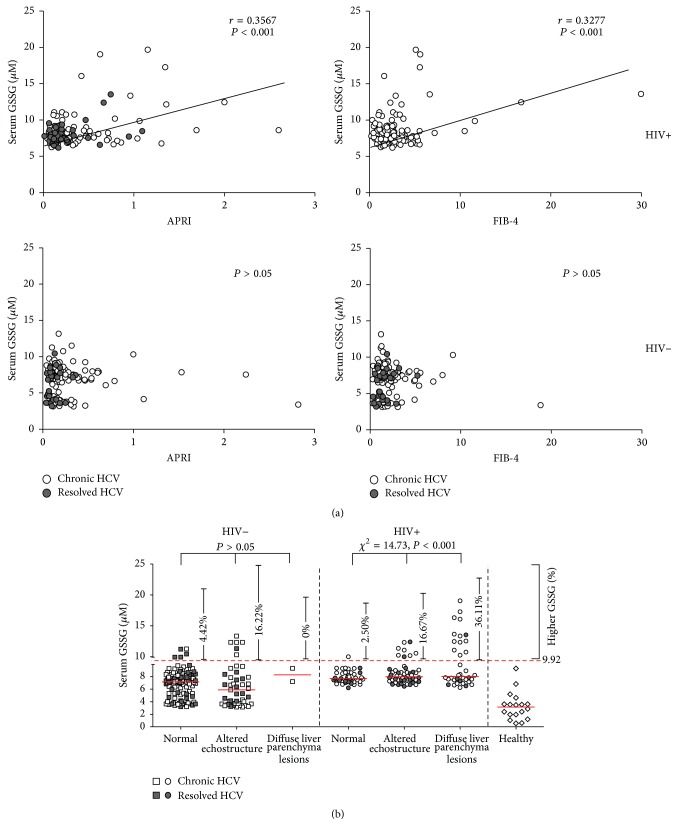
Association between aggravated liver damage in HCV/HIV-coinfected individuals and higher serum GSSG concentrations. (a) Correlation between APRI (left panel) and FIB-4 (right panel) and serum GSSG concentration in HIV-positive and HIV-negative patients. Correlations of GSSG with APRI and FIB-4 were evaluated by Spearman correlation coefficient. (b) Serum GSSG concentrations in HIV-positive (right panel) and HIV-negative (left panel) patients were compared among patients with different liver ultrasound manifestations (normal, altered echostructure, and diffuse liver parenchymal lesions). The upper level (9.92 *μ*M) of normal serum GSSG was set as the mean plus three times the standard deviation of healthy controls. Trends in GSSG levels among patients categorized by liver ultrasound manifestations were analyzed by Chi-squared tests. All *P* values were two-tailed, with *P* < 0.05 considered statistically significant.

Furthermore, our data showed that serum GSSG was positively correlated with APRI (*r* = 0.3567, *P* < 0.001) and FIB-4 (*r* = 0.3277, *P* < 0.001) indexes in HIV^pos^ groups, but not in HIV^neg^ counterparts (*P* > 0.05) (Figure 4(a)). The relationship of serum GSSG with different liver ultrasound manifestations in the presence or absence of HIV infection was also evaluated. As shown in Figure 4(b), the upper level of normal serum GSSG (9.92 *μ*M) was set as mean plus three times standard deviation based on the values of healthy controls. The results of Chi-square tests showed that serum GSSG was associated with altered echostructure or diffuse liver parenchyma lesions in HIV^pos^ subjects (*χ* ^2^ = 14.73, *P* < 0.001) but not in HIV^neg^ individuals (*P* > 0.05). These results indicated that HIV infection contributed at least partially to liver damages in the context of HCV infection, which to some extent ascribed to elevated oxidative stress.

## 4. Discussion

Some studies demonstrated that oxidative stress and liver injury were more pronounced in HIV/HCV coinfection than HIV monoinfection [19, 21]. However, few studies focused on the comparison of oxidative stress between HIV/HCV coinfection and HCV monoinfection. In this study, we demonstrated that HIV/HCV coinfection induced aggravated liver damages compared to HCV monoinfection and a linkage between HIV-induced oxidative stress and the higher incidence rate of advanced liver disease was found in coinfected patients.

In contrast to data from erythrocytes of HCV-infected patients [25] and to our expectations, we found that the antioxidant markers, reduced GSH and GSH-Px, were elevated in patients infected with HIV alone but not in those infected with HCV alone. This discrepancy may ascribe to different sample type used in different studies (serum versus erythrocyte). Serum samples are easier to assay and are likely more accurate in reflecting the status of circulating free radicals. The consistent patterns of GSH-Px and reduced GSH concentrations in subjects infected with HIV and/or HCV support the validity of assaying these markers in serum samples.

HIV-infected individuals had lower levels of intracellular glutathione in T-cell subsets, with CD4+/CD8+ T-cells having higher levels of intracellular glutathione selectively lost as HIV infection progressed [26, 27]. Our study demonstrated that both serum oxidants (MDA and GSSG) and antioxidants (GSH-Px and reduced GSH) were elevated in chronic HIV/HCV-coinfected patients, but serum zinc was not. The enhanced destruction of lymphocytes and erythrocytes in patients chronically infected with HIV may result in the release of MDA, GSH-Px, GSSG, and reduced GSH. This may partially explain why serum antioxidant indicators were not increased in chronic HCV infection but were markedly increased in HIV/HCV coinfection. However, as an oxidized GSH, serum GSSG was found to be a superior indicator of oxidative stress, during both HCV and HIV infection, as it was increased in chronically HCV-infected, HCV-resolved, and HIV/HCV-coinfected patients.

Hepatotoxicity is a great concern in HIV/HCV-coinfected patients. High HCV-associated morbidity and mortality are common among coinfected patients, even those treated with HAART [28, 29]. This study found that HCV-associated ESLD was a greater cause of death in HIV/HCV-coinfected patients treated with HAART than in HCV-monoinfected patients. It is unclear, however, whether antiretroviral therapy has a negative or positive effect on liver hepatitis in HIV/HCV-coinfected patients [30]. HAART-driven immune reconstitution may retard HIV replication in lymphocytes resident in the liver, thereby theoretically contributing to the ameliorative microenvironment of the liver. Indeed, several retrospective studies have reported a slower progression of liver damage in HAART-treated patients [31, 32]. By contrast, the restoration of immune function may enhance preexisting anti-HCV-specific immune responses in HAART-treated HIV/HCV-coinfected patients. In addition, HAART-associated drug-induced liver injury (DILI) may worsen HCV-related hepatitis in a small number of patients, especially those treated with didanosine, nevirapine, and efavirenz [31–35]. The findings presented in this study clearly showed that HIV coinfection increased serum levels of markers of oxidative stress, both in patients with chronic HCV infection and in HCV resolvers. Although serum antioxidant and oxidant parameters did not correlate with CD4+ T-cell counts in HIV-coinfected patients (data not shown), our data indicated that higher reduced GSH concentrations appeared in HIV-infected patients with higher CD4+ T-cell compared to patients with lower CD4+ T-cell, suggesting that antiretroviral therapy (ART) played a beneficial role in preventing aggravation of oxidative stress status. Similarly, an improvement in the abnormal GSH redox status was found in HIV patients receiving successful ART [36] and HIV infection without ART was associated with lower reduced GSH levels in the lung [37]. We speculated that higher oxidative stress status in HIV/HCV-coinfected patients might ascribe to complicated reasons, including immune system impairment, direct HIV/HCV cytotoxicity, iron load, and/or treatment with HAART.

Ultrasound examination of our patients clearly indicated that aggravated liver parenchymal lesions were more frequent in HIV-positive than in HIV-negative individuals, both in chronic HCV carriers and in HCV resolvers. These patients were not assessed by transient elastography, however, because medical resources at the local medical care were limited at the time the study was initiated. As an alternative, stages of liver fibrosis in these patients were determined using two noninvasive indices of fibrosis, APRI and FIB-4, which have been shown to be valid in evaluating liver fibrosis in numerous studies [23, 38–44]. For example, FIB-4 scores were found to strongly correlate with biopsy-determined stages of HCV-associated liver fibrosis in a large observational cohort of chronic HCV patients [45]. We also found that these two indicators were strongly consistent and correlated with each other in both HCV-monoinfected and HIV/HCV-coinfected patients (data not shown). Both APRI and FIB-4 were higher in HIV-positive than in HIV-negative HCV-infected patients. Interestingly, correlations between APRI/FIB-4 and serum GSSG were observed only in HIV-coinfected patients, not in HIV-negative chronic HCV carriers and HCV resolvers. Similar association was also found between serum GSSG and poorer liver ultrasound manifestation in HIV-infected individual. These findings indicated that, in HIV/HCV-coinfected patients, worsening liver fibrosis status may be associated with HIV infection and may correlate, at least in part, with higher levels of oxidant markers, such as GSSG. Conversely, higher serum GSSG levels were consistent with abnormal levels of serum ALT/AST in HIV/HCV-coinfected but not in HCV-monoinfected patients. Interestingly, a recent study demonstrated that active viral replication induces strong oxidative stress in HIV-1 infected cell lines and a moderate increase of oxidative stress is sufficient to switch HIV-1 from latency to reactivation, which indicated that HIV replication can be a crucial cause of oxidative stress and may contribute to concomitant liver damage, irrespective to HAART [46]. We speculated that higher concentrations of circulating prooxidative components induced by long-term HIV replication* in vivo* may continuously and adversely influence the hepatic microenvironment in HCV-infected patients, resulting in acceleration of the occurrence of fibrosis and cirrhosis, such as poorer liver ultrasound manifestation and elevated APRI/FIB-4 scores in this study. Although HIV induction of a greater degree of oxidative stress was not the sole cause of greater liver damage initially due to HCV infection, it was likely to be at least partially responsible.

Near 100% of the HIV-positive patients in our cohort were also infected with HCV, or were at least positive for anti-HCV antibodies. As only a very small number of HIV-monoinfected patients negative for anti-HCV were found in this village, their number was too small for statistical analysis and they were not included in this study.

## 5. Conclusions

In conclusion, HCV/HIV-coinfected patients showed accelerated liver damage compared with HCV-monoinfected patients in the present study. This aggravated liver damage was likely due to increased oxidative stress, mainly induced by HIV coinfection.

## Supplementary Material

Supplementary material includes four supplementary Figures and one supplementary Table.
Figure S1 shows Flow diagram for subjects recruited to this study. Figure S2 introduces comparison of serum ALT and AST enzyme activities among five groups containing HIV-negative chronic HCV carriers, HIV-positive chronic HCV carriers, HIV-negative HCV resolvers, HIV-positive HCV resolvers, and healthy controls. Figure S3 indicates APRI and FIB-4 scores are higher in HIV-positive than in HIV-negative subjects. Figure S4 shows that negative correlations were found between CD4+ T-cell counts and APRI/FIB-4 scores in HIV-positive HCV resolvers.
Table S1 shows the clinical characteristics of the 158 HCV-monoinfected and 124 HIV/HCV-coinfected patients enrolled in 2009 in this study.

## Figures and Tables

**Table 1:** Mortality rates in HCV-monoinfected and HCV/HIV-coinfected patients from 2005 to 2009.

Variable	HCV monoinfection	HCV/HIV coinfection	*P* value
Total patients in 2005 (*n*)	223	140	
Total deaths [*n* (% of total patients)]	7 (3.14%)	29 (20.7%)	<0.001
AIDS-related deaths [*n* (% of total death)]	0 (0%)	17 (58.6%)	0.005
Non-AIDS-related deaths [*n* (% of total death)]	7 (100%)	12 (41.4%)	0.005
ESLD-related deaths [*n* (% of non-AIDS-related death)]	1 (14.3%)	8 (66.7%)	0.027
Other causes of deaths [*n* (% of non-AIDS-related death)]	6 (85.7%)	4 (33.3%)	0.027

HCV: hepatitis C virus; HIV: human immunodeficiency virus; ESLD: end-stage liver disease.

**Table 2:** Liver ultrasound manifestations in HIV-positive and HIV-negative patients with/without chronic HCV or resolved HCV.

Liver ultrasound manifestation	HIV^neg^ chronic HCV	HIV^pos^ chronic HCV	*P* ^a^ value	HIV^neg^ resolved HCV	HIV^pos^ resolved HCV	*P* ^b^ value
Total patients (*n*)	102	76		56	48	

Echostructure			<0.001			<0.001
Normal [*n*, (%)]	73 (71.6%)	24 (31.6%)		40 (71.4%)	16 (33.3%)	
Altered echostructure [*n*, (%)]	27 (26.5%)	22 (28.9%)		16 (28.6%)	26 (54.2%)	
Diffuse liver parenchyma lesions [*n*, (%)]	2 (2.0%)	30 (39.5%)		0	6 (12.5%)	
Others						
Hepatomegaly [*n*, (%)]	1 (1.0%)	2 (2.6%)	0.790	0	1 (2.1%)	0.462
Fatty liver [*n*, (%)]	7 (6.9%)	6 (7.9%)	0.794	4 (7.1%)	4 (8.3%)	1.00

HCV: hepatitis C virus; HIV: human immunodeficiency virus; *P* ^a^ comparisons between HIV-negative and HIV-positive groups with chronic HCV infection; *P* ^b^ comparisons between HIV-negative and HIV-positive groups with resolved HCV.

## Abbreviations

tGSH: Total glutathione
GSSG: Oxidized glutathione
MDA: Malondialdehyde
GSH-Px: Glutathione peroxidase
HCV: Hepatitis C virus
HIV-1: Human immunodeficiency virus-1
FBDs: Former blood donors
DCs: Dendritic cells
NRTIs: Nucleoside reverse transcriptase inhibitors
NNRTIs: Nonnucleoside reverse transcriptase inhibitors
ALT: Alanine aminotransferase
AST: Aspartate aminotransferase
*γ*-GT: *γ*-Glutamyl transpeptidase
APRI: Aspartate aminotransferase to platelet ratio index
FIB-4: Fibrosis index based on four factors
ESLD: End-stage liver disease
HAART: Highly active antiretroviral therapy.
