# Supplementary material for: Liver Damage in Patients with HCV/HIV Coinfection Is Linked to HIV-Related Oxidative Stress
Source: Oxid Med Cell Longev. 2016 Jan 10;2016:8142431. doi: 10.1155/2016/8142431 (PMC4736998; doi:10.1155/2016/8142431)
Supplement: Supplementary file 1 — Supplementary material includes four supplementary Figures and one supplementary Table. Figure S1 shows Flow diagram for subjects recruited to this study. Figure S2 introduces comparison of serum ALT and AST enzyme activities among five groups containing HIV-negative chronic HCV carriers, HIV-positive chronic HCV carriers, HIV-negative HCV resolvers, HIV-positive HCV resolvers, and healthy controls. Figure S3 indicates APRI and FIB-4 scores are higher in HIV-positive than in HIV-negative subjects. Figure S4 shows that negative correlations were found between CD4+ T-cell counts and APRI/FIB-4 scores in HIV-positive HCV resolvers. Table S1 shows the clinical characteristics of the 158 HCV-monoinfected and 124 HIV/HCV-coinfected patients enrolled in 2009 in this study. [file 8142431.f1.docx]

## Supplementary materials for manuscript entitled

## Liver damage in patients with HCV/HIV coinfection is linked to HIV-related oxidative stress

## Xiangbo Huang, Hua Liang, Xueying Fan, Liyan Zhu, Tao Shen

**Figure S1.** Flow diagram for subjects recruited to this study.

**
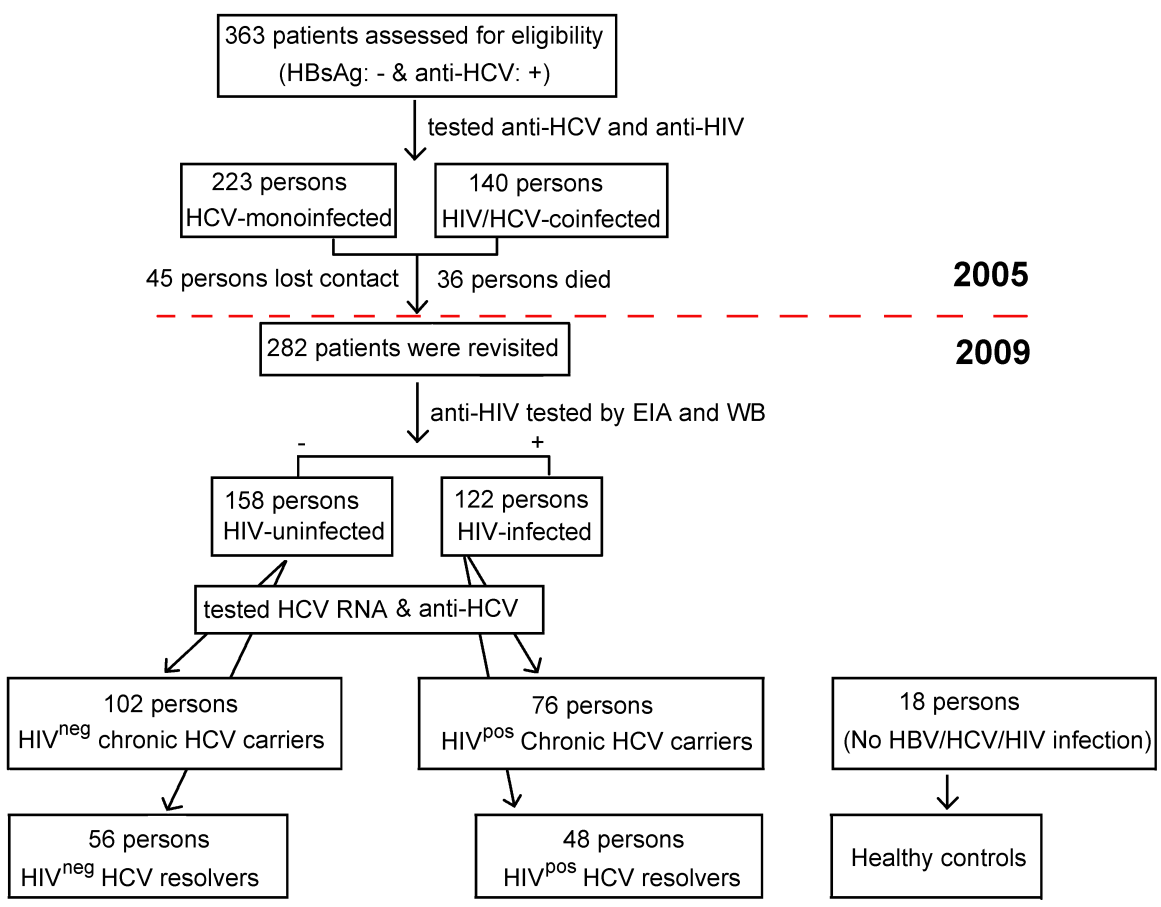
**

**Figure S2.** Serum ALT (○) and AST (●) enzyme activities in HIV-negative chronic HCV carriers, HIV-positive chronic HCV carriers, HIV-negative HCV resolvers, HIV-positive HCV resolvers, and healthy controls. The percentages of patients in each group with abnormal serum ALT or AST activities (>40 IU/L) are indicated. The median value for each group is shown as a vertical bar. All p-values were two-tailed, with p<0.05 considered statistically significant.

**
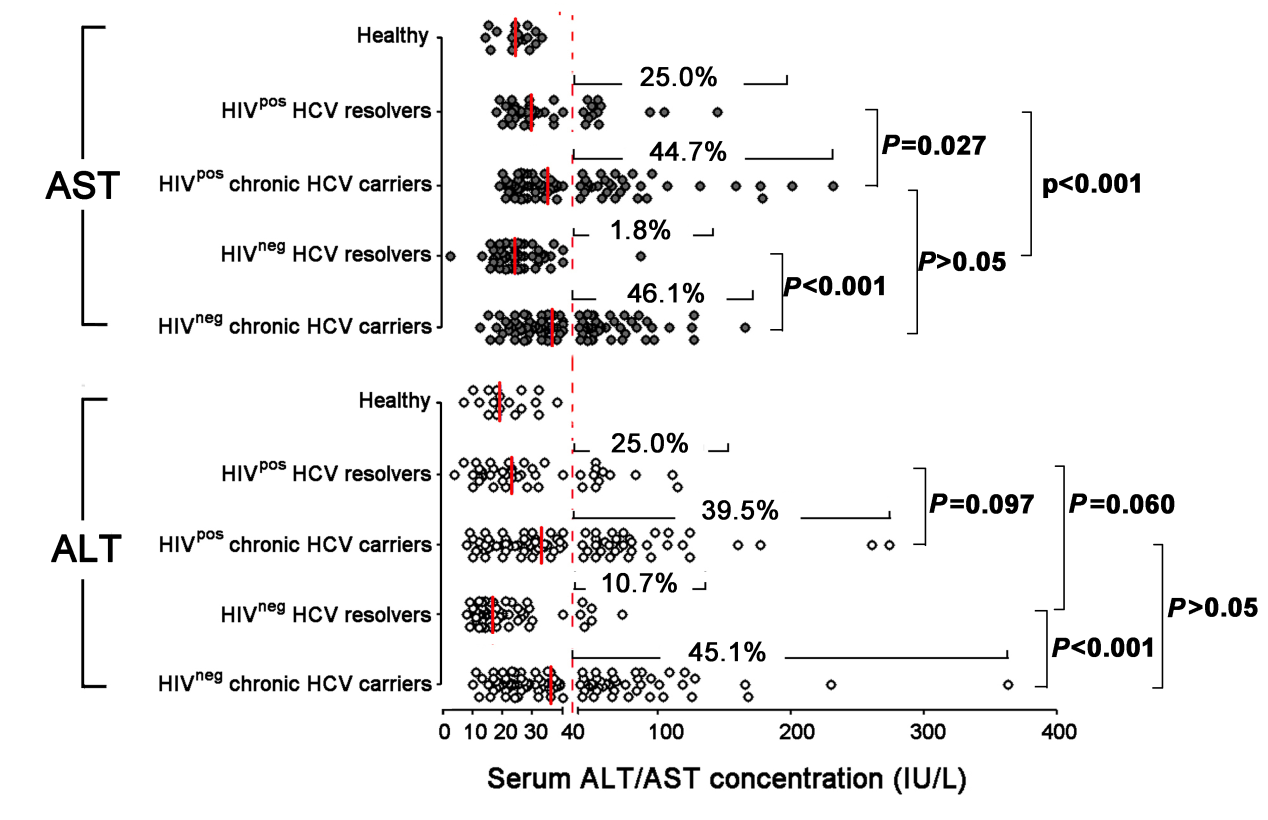
**

**Figure S3.** APRI and FIB-4 scores are higher in HIV-positive than in HIV-negative subjects. APRI (a) and FIB-4 (b) indexes were determined in HIV-negative chronic HCV carriers (●), HIV-positive chronic HCV carriers (○), HIV-negative HCV resolvers (■), HIV-positive HCV resolvers (□), and healthy controls (◇). APRI (c) and FIB-4 (d) indexes were determined in HIV-positive subjects with different liver ultrasound manifestations (normal, altered echostructure, and diffuse liver parenchymal lesions). The median value for each group is indicated as a red line. Unpaired nonparametric t tests were used for between-group comparisons. All p-values were two-tailed, with p<0.05 considered statistically significant.

**
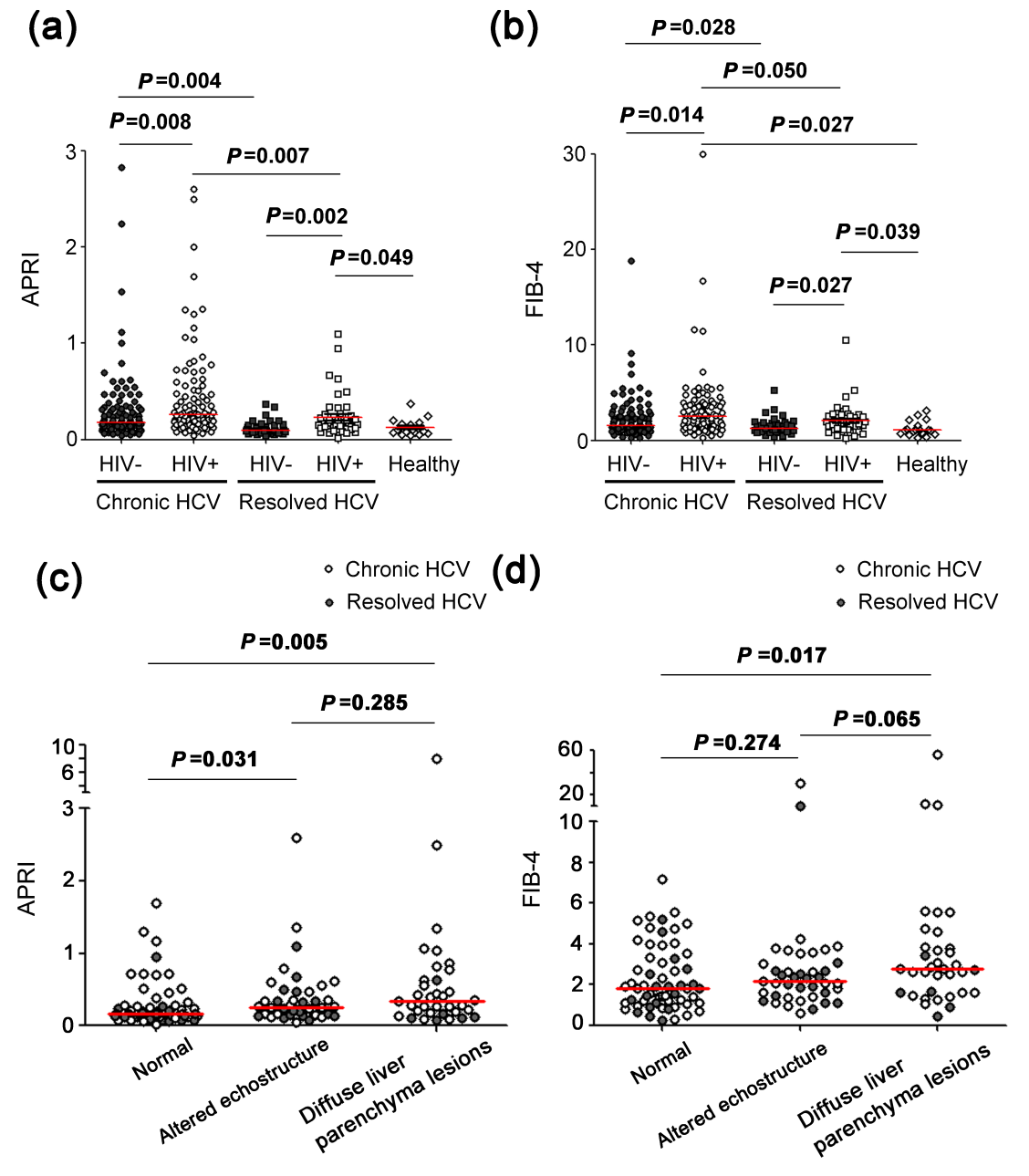
**

**Figure S4**. Significant negative correlations were found between CD4+ T-cell counts and APRI/FIB-4 scores in HIV-positive HCV resolvers (a), but not in HIV-positive chronic HCV carriers (b). Correlations between groups were conducted using spearman’s rank-correlation test. All p-values were two-tailed, and p<0.05 was considered statistically significant.

**
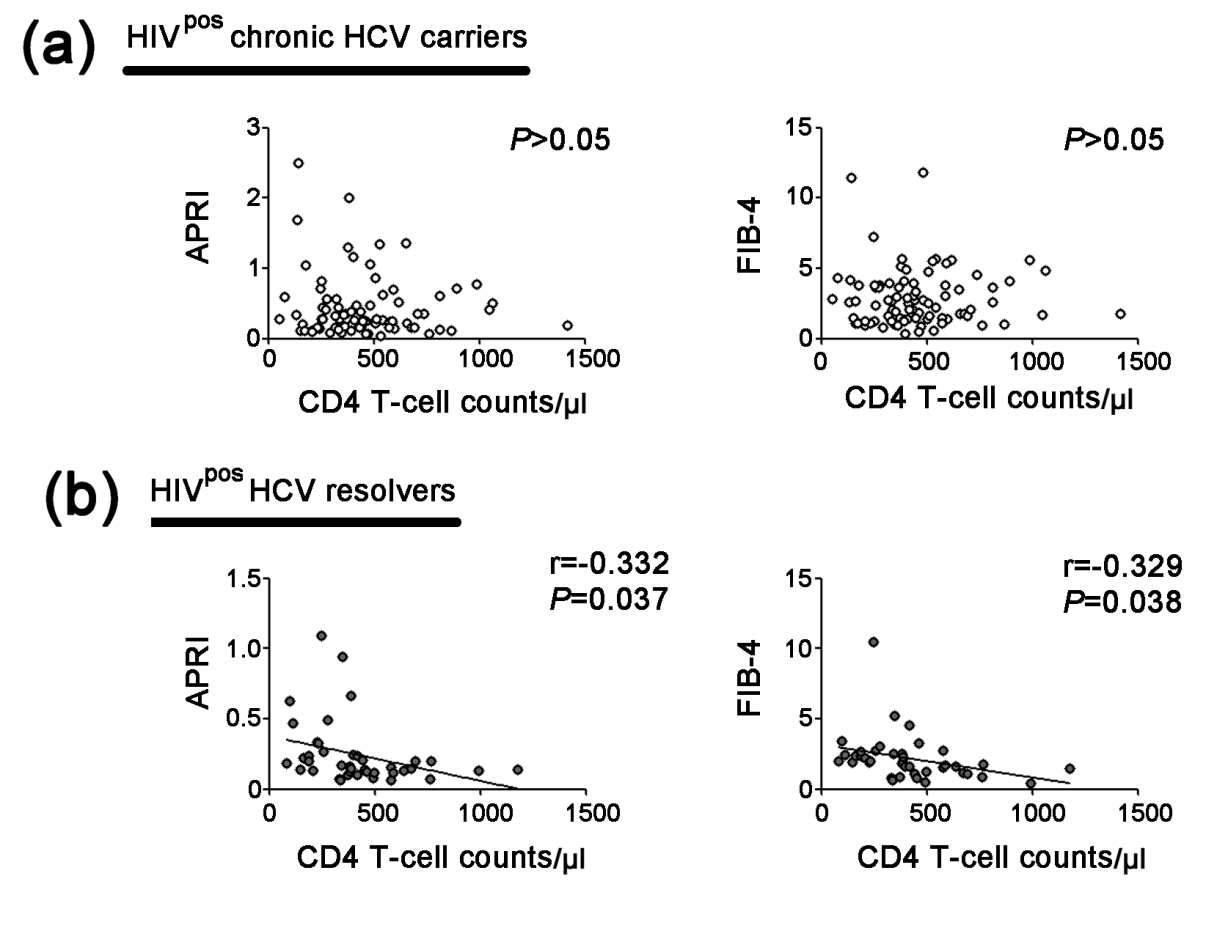
**

Table S1. Clinical characteristics of the 158 HCV-monoinfected and 124 HIV/HCV-coinfected patients enrolled in 2009.

| **Characteristic** | **HCV-monoinfected (n=158)** | | **HIV/HCV-coinfected (n=124)** | |
| --- | --- | --- | --- | --- |
| **HCV status, (n)** | **Chronic carriers** | **Resolved** | **Chronic carriers** | **Resolved** |
| **Gender, n(%)** |  | | | |
| ***Male*** | 37 (36.3) | 7 (12.5) | 23 (30.3) | 21 (43.7) |
| ***Female*** | 65 (63.7) | 49 (87.5) | 53 (69.7) | 27 (56.3) |
| **Age (years)** | 52(42-59) | 48 (39-55) | 51 (41-57) | 49 (40-56) |
| **HBsAg** | - | - | - | - |
| **anti-HIV** | - | - | + | + |
| **CD4+ T-cell counts** | 815 (588-1044) | 875 (335-950) | 417 (287-554) | 395 (251-566) |
| **CD8+ T-cell counts** | 551 (442-854) | 625(415-890) | 952 (652-1186) | 975 (710-1258) |
| **HIV RNA (copies/mL) , n(%)** |  |  |  |  |
| ***>1000*, n(%)** | N.A | N.A | 27 (35.5) | 19 (39.6) |
| ***<1000*, n(%)** | N.A | N.A | 49 (64.5) | 29 (60.4) |
| **HIV genotype, n(%)** |  |  |  |  |
| ***B'*** | N.A | N.A | 46 (60.5) | 28 (58.3) |
| ***Others*** | N.A | N.A | 0 (0) | 0 (0) |
| ***Undetectable*** | N.A | N.A | 30 (39.5) | 20 (41.7) |
| **anti-HCV S/CO value** | 14.7 (13.7-15.6) | 8.5 (4.6-10.9) | 14.1 (12.8-15.3) | 7.9 (4.8-11.0) |
| **HCV RNA(log_10_ IU/mL)** | 6.2 (5.8-6.6) | - | 6.4 (5.8-6.8) | - |
| **HCV genotype, n(%)** |  |  |  |  |
| ***1b*** | 65 (63.7) | N.A | 34 (44.7) | N.A |
| ***2a*** | 37 (36.3) | N.A | 42 (55.3) | N.A |
| ***Others*** | 0 (0) |  | 0 (0) |  |
| **Blood routine** |  |  |  |  |
| ***WBC (10^9^/L)*** | 6.5 (4.8-7.8) | 6.3 (4.7-7.9) | 5.2 (4.8-6.5) | 5.3 (4.7-6.0) |
| ***RBC (10^12^/L)*** | 5.2 (4.8-5.4) | 5.2 (4.9-5.4) | 4.1 (4.2-5.2) | 4.3 (4.1-5.0) |
| ***Hemoglobin (g/L)*** | 142 (130-152) | 143 (132-155) | 115 (108-132) | 122 (108-136) |
| ***Platelet (10^9^/L)*** | 238(195-282) | 225 (194-258) | 146.0 (104-182) | 139 (95-186) |
| **Biochemistry analysis** |  |  |  |  |
| ***ALT (IU/L)*** | 39 (25-61) | 24 (15-41) | 38 (21-65) | 30 (18-44) |
| ***AST(IU/L)*** | 37 (30-52) | 21 (18-30) | 38 (27-62) | 24 (14-36) |
| ***Total protein (g/L)*** | 78 (73-80) | 76 (72-78) | 77 (74-82) | 77 (70-81) |
| ***Albumin (g/L)*** | 44 (40-47) | 45 (41-48) | 44 (42-48) | 43 (40-47) |
| ***Total bilirubin (μmol/L)*** | 14.1 (11.1-16.0) | 12.3 (10.5-16.1) | 13.9 (10.6-15.6) | 12.7 (10.2-14.8) |
| ***Direct bilirubin (μmol/L)*** | 4.3 (3.4-5.2) | 4.2 (3.2-5.2) | 4.4 (3.4-5.3) | 4.3 (3.4-5.5) |
| **BMI, mean (SD)** | 23.2 (21.2-25.5) | 23.0 (20.6-24.6) | 22.6 (20.8-24.2) | 22.8 (21.0-24.5) |
| **Fatty liver, n(%)** |  |  |  |  |
| ***none*** | 95 (93.1) | 52 (92.9) | 70 (92.1) | 44 (91.7) |
| ***Low-grade*** | 7 (6.9) | 4 (7.1) | 6 (7.9) | 4 (8.3) |
| ***Medium-grade*** | 0(0) | 0(0) | 0(0) | 0(0) |
| **Liver cancer, n(%)** | 0(0) | 0(0) | 0(0) | 0(0) |
| **Hypertension, n(%)** | 9(12.33) | 13(17.8) | 10(15.15) | 14(21.2) |
| **Diabetes, n(%)** | 0 (0) | 1 (1.78) | 0 (0) | 2(4.17) |
| **Hyperlipidemia, n(%)** | 4 (3.92) | 5 (8.93) | 5(6.58) | 3 (6.25) |
| **HAART, n(%)** |  |  |  |  |
| ***Occasional*** | N.A | N.A | 0 (0) | 0 (0) |
| ***Intermittent*** | N.A | N.A | 11 (14.5) | 7(14.6) |
| ***Regular*** | N.A | N.A | 65 (85.5) | 41(85.4) |
| **Duration of HAART (years)** | N.A | N.A | 6.5 (4.8-7.5) | 6.5 (5.0-7.5) |

Data are presented as median (interquartile range) unless otherwise indicated. HIV, human immunodeficiency virus; HCV, hepatitis C virus; RBC, red blood cells; WBC, white blood cells; ALT, alanine aminotransferase; AST, aspartate aminotransferase; FBDs, former blood donors; HAART, highly active antiretroviral therapy; BMI, body mass index, calculated as the weight in kilograms divided by the square of height in meters; +, positive; -, negative; N.A., not available.
